# Invisible work: Child work in households with a person living with HIV/AIDS in Central Uganda

**DOI:** 10.1080/17290376.2017.1379429

**Published:** 2017-10-11

**Authors:** Julie Abimanyi-Ochom, Brett Inder, Bruce Hollingsworth, Paula Lorgelly

**Affiliations:** ^a^ PhD, Research Fellow at Deakin Health Economics, Centre for Population Health Research, Deakin University, Geelong, Victoria 3220, Australia; ^b^ PhD, Professor at the Centre for Health Economics, Monash University, Clayton, Victoria 3800, Australia; ^c^ PhD, Professor at the Division of Health Research, Lancaster University, Lancaster LA1 4YT, UK; ^d^ PhD, Associate Professor at the Centre for Health Economics, Monash University, Clayton, Victoria 3800, Australia

**Keywords:** Central Uganda, unpaid child work, domestic work, family farm work, HIV/AIDS households, Sub-Saharan Africa, J13, I10, D13, Ouganda central, travail domestique, travail sur la ferme familiale, ménages VIH/SIDA, tâches en dehors du marché du travail, Afrique subsaharienne

## Abstract

*Background*: HIV/AIDS has led to increased mortality and morbidity, negatively impacting adult labour especially in HIV/AIDS burdened Sub-Saharan Africa. There has been some exploration of the effects of HIV/AIDS on paid child labour, but little empirical work on children’s non-paid child work. This paper provides quantitative evidence of how child and household-level factors affect children’s involvement in both domestic and family farm work for households with a person living with HIV/AIDS (PLWHA) compared to non-PLWHA households using the 2010/2011 Centre for Health Economics Uganda HIV questionnaire Survey. *Method:* Descriptive analysis and multivariate logistic modelling is used to explore child and household-level factors that affect children’s work participation. *Results*: This research reveals greater demands on the labour of children in PLWHA households in terms of family farm work especially for boys. Results highlight the expected gendered social responsibilities within the household space, with girls and boys engaged more in domestic and family farm work, respectively. Girls shared a greater proportion of household financial burden by working more hours in paid work outside the household than boys. Lastly, the study revealed that a household head’s occupation increases children’s participation in farm work but had a partial compensatory effect on their involvement in domestic work. Wealth and socio-economic standing is no guarantee to reducing child work. *Conclusion*: Children from PLWHA households are more vulnerable to child work in family farm work especially boys; and girls are burdened beyond the household space through paid work. Differing perspectives and solutions need to consider the contextual nature of child work.

## Résumé

Le VIH/SIDA a mené à des taux de mortalité et morbidité accrus, avec des impacts négatifs pour la capacité de travail des adultes, surtout en Afrique subsaharienne qui souffre tout particulièrement du fardeau du VIH/SIDA. Quelques recherches ont déjà été menées sur les effets du VIH/SIDA sur le travail rémunéré des enfants, mais il existe fort peu d’enquêtes empiriques sur le travail volontaire des mineurs (des travaux qui restent en dehors du marché du travail), tels que du travail dans la maison familiale ou sur la ferme familiale. Cet article fournit des données quantitatives sur les facteurs (au niveau de l’enfant et au niveau du ménage) qui exercent une influence sur l’implication des enfants dans ces tâches domestiques et agricoles. L’article compare des enfants provenant des ménages avec une Personne Vivant avec VIH (PVVIH) avec des enfants des ménages non-PVVIH, en s’appuyant sur le Sondage du Centre pour l’Economie de la Santé d’Ouganda (Centre for Health Economics Uganda HIV Survey) de 2010/2011. L’article déploie des analyses descriptives aussi bien que la modélisation logistique multivariée pour explorer des facteurs (au niveau de l’enfant et du ménage) ayant une influence sur la participation des enfants au travail. Les recherches montrent que les attentes sur des enfants qui viennent des ménages PVVIH sont plus élevées lorsqu’il s’agit de travailler sur la ferme familiale, et ceci surtout pour les garçons. Les résultats soulignent les responsabilités sociales sexuées attendues au sein du ménage, les filles engagées plus au travail domestiquetandisque les garçons travaillent plus sur la ferme familiale. Les filles endossaient une proportion plus élevée du fardeau financier familial parce qu’elles avaient plus d’heures de travail payé en dehors du ménage que les garçons. En dernier lieu, l’étude montre que l’occupation du chef de famille augmente la participation des enfants au travail sur la ferme familiale, mais exerce un effet compensatoire partiel sur leur implication dans le travail domestique. Les résultats suggèrent que la richesse et le statut socio-économique ne garantit pas une réduction d’heures de travail pour l’enfant, donc il faut que des solutions et des perspectives différentes considèrent la nature contextuelle du travail des enfants.

## Introduction

HIV/AIDS adds a new dimension to the child labour problem (ILO, 2003), especially in Sub-Saharan Africa (SSA) where the burden of the disease is greatest. Children residing in households that have a person living with HIV/AIDS (PLWHA) are particularly vulnerable given that HIV/AIDS affects adults in their productive prime, often affecting their income (Evans, 2013; Taraphdar et al., 2011).

It is argued that HIV/AIDS impacts child labour in several ways: by increasing the number of vulnerable children, especially orphans that have lost at least one parent to HIV/AIDS; by placing an inequitable burden on female children, who often have to provide care and household services for the entire family when a parent becomes ill or dies; and by putting pressure on children to work to assist their families to obtain a livelihood and survive (Daniel, 2011; Desmond, 2009; Engle, 2008; Evans, 2012; ILO, 2003; Nyamukapa & Gregson, 2005; Rau, 2002; Tumushabe, 2000).

Global estimates indicate a reduction in new infections among children and a reduction in AIDS-related deaths overall (Foster, Laugharn, & Wilkinson-Maposa, 2010; UNAIDS, 2010, 2013, 2014). This is partly attributed to antiretroviral therapy (ART) which has been shown to restore health and improve health outcomes even in resource constrained countries in SSA (Seeley et al., 2009; Song et al., 2007; UNAIDS, 2013, 2016). However, despite the decline, HIV/AIDS continues to impact communities and households in SSA, through its devastating impact on quality of life through poor health, impact on school performance and reduced life-expectancy (Boutayeb, 2009; Bukusuba, Kikafunda, & Whitehead, 2007; Daniel, 2011; Grogan, 2008; Maguire, McNally, Britton, Werth, & Borges, 2008; Richter, 2004; Tumushabe, 2000; UNAIDS, 2010; UNAIDS, UNICEF, & USAID, 2004; UNICEF & UNAIDS, 2005).

In Africa, strong family and kinship networks function as the traditional social support systems (‘safety net’) in times of need. This extended family support system has been reported to form the basis of orphan care and education in SSA including East Africa. However, with the changes in labour migration, demographic changes, urbanisation and the advent of HIV/AIDS, the extended family support system has been weakened and overburdened. HIV/AIDS has eroded the material and emotional resources available to communities and families affected by the epidemic thereby limiting access to formal and informal safety nets.

In many societies in SSA, socio-cultural norms, levels of poverty and negative impact of HIV/AIDS mean that most children are expected to participate in work from an early age. Consequently, it is quite common for children to work within their family, extended family and the community (Evans, 2010; Skovdal, Campbell, & Onyango, 2013). Responsibilities may include paid and unpaid work and are normally valued as an integral part of children’s informal education and socialising in the family and community (Evans & Skovdal, 2016). Children are involved in both productive and social reproductive activities that are normally defined by gender. Girls in SSA often undertake domestic chores within the household including cooking, cleaning laundry, fetching water and child care, while boys engage more in activities outside the household including livestock rearing, unpaid in-kind-payment work and paid work (Evans, 2010; Evans & Skovdal, 2016; Miller, 2005; Ridge, 2006). It is common for children’s roles and responsibilities to be differentiated by gender, age, sibling birth order, household composition and intergenerational relations (Punch, 2001; Such & Walker, 2004). Table 1 outlines the different dimensions of care work undertaken by children and young adults in Africa as adapted from Evans and Becker (2009) and Evans (2010, 2014).

**Table 1.** Dimensions of children’s and young people’s care work in Africa.

Caring activity	Examples
*Household chores*	Cooking, washing dishes, sweeping, cleaning and tidying, fetching water and firewood, laundry, heating water for baths, shopping, cultivating food for consumption, tending livestock, cutting wood, running errands
*Health care*	Reminding parent/sibling/relative to take medication, giving and collecting medication, accompanying them to hospital and providing care while in hospital, assisting with mobility, preparing special nutritional food, cleaning, treating and dressing sores, infections and wounds, massaging the body
*Personal care*	Washing/bathing parent/relative, assisting to eat, dress and use the toilet
*Child care*	Getting siblings ready for school, bathing siblings, supervision, resolving arguments and conflict between siblings, help with school work
*Emotional support*	Talking and comforting parent/sibling/ relative, giving advice and guidance, being there for them
*Self-care*	Personal care of self, taking medication, getting ready for school, private study, personal development, training, developing life skills and livelihood strategies
*Income generation activities*	Cultivating crops and produce for sale, rearing livestock, casual agricultural and construction work, fishing, working in a factory, shop or bar, selling produce, cooked food, charcoal and other goods, domestic work, running errands for neighbours, begging
*Household management*	Allocating tasks, paying school contributions, organising school/vocational training, reminding parent/sibling/relative about appointments, paying bills and resolving financial problems, budgeting, future planning and decision-making
*Community engagement*	Maintaining social networks, seeking support from and cooperating with relatives, neighbours, friends, NGOs, members of faith community, participating in neighbourhood, school, faith community, youth and NGO meetings, activities, celebrations and events

Source: Evans and Becker (2009, p. 130) and Evans (2010, p. 1481, 2014, p. 1898).

However, with the advent of HIV/AIDS, the traditional expectations have been replaced by the increased reliance on children for labour due to lack of alternative support (Evans, 2010; Kesby, Gwanzura-Ottemoller, & Chizororo, 2006; Kuo & Operario, 2010; Skovdal, Magutshwa-Zitha, Campbell, Nyamukapa, & Gregson, 2013; Tumushabe, 2000; UNICEF, 2000). Children in households impacted by HIV/AIDS are shown to be involved in a wide range of caring responsibilities, with such responsibilities evidenced to go beyond the usual expectations of children’s household responsibilities (Evans, 2010; Robson, Ansell, Huber, Gould, & van Blerk, 2006; Skovdal, Ogutu, Aoro, & Campbell, 2009).

Evans (2010, 2014) demonstrates the level of children’s care responsibilities using the continuum of young caregiving. Children’s informal caring is conceptualised ranging from ‘caring about’ to ‘caring for’ whereby care through every day practices of care in terms of very intimate proximity refers to ‘caring for’ while ‘caring about’ is less direct and usually performed at a distance (Barnett & Land, 2007; Evans, 2014).

Children residing in households affected by HIV/AIDS can be placed at the ‘high’ end of the caring continuum given their caring responsibilities for the PLWHA whenever they become ill (See Supplementary Figure 1). Most children around the world are involved in low levels of caring while a small proportion, are involved in much higher levels of care due to different life circumstances for example having a close relative living with HIV/AIDS (Evans, 2014; Lane, Cluver, & Operario, 2015; Robson et al., 2006). Children’s caring roles have been shown to shift and change over time and place (Evans & Becker, 2009). Therefore, for households with a PLWHA, children’s position on the continuum would be versatile since children’s responsibilities would change over time and space. This will depend on variation in a parent’s health and need for assistance given changing access to formal and informal safety nets that may reduce on children’s caring responsibilities (Evans, 2014).

Regardless of the important contribution that children make to their family and society, children carrying out such domestic tasks within the family are often not regarded as economically active. The ILO definition of employed children excludes such work rendering children’s work contributions invisible (Bhukuth, 2008; de Lange, 2009; Evans, 2014; Evans & Skovdal, 2016). Becker (2007) highlights how international child welfare concerns ignore children’s social reproductive work within the family within development policy and planning by focusing more on visible forms of productive work (Becker, 2007).

Nevertheless, children working within the household have been shown to be vulnerable as a result of their caring responsibilities. Caring responsibilities for children from households with a PLWHA may lead to negative outcomes due to their care work being located at the high end of the caregiving continuum, complicated by widespread poverty and missing support systems. Young people have reported experiencing mistreatment in terms of bullying, harassment and stigma as a consequence of their caring roles and HIV/AIDS-related stigma (Evans, 2014). Caring roles compete with children’s education time, disrupting school attendance. This impacts their performance at school and long-term employment opportunities and hence future welfare (Evans, 2014; Evans & Becker, 2009; Hazarika & Sarangi, 2008; Skovdal et al., 2013). The magnitude of the negative impact of caring roles is difficult to ascertain due to the confounding impact of poverty, social exclusion and limitations of the disadvantaged households that these children belong (Evans, 2014; Evans & Becker, 2009).

There is evidence that children affected by HIV/AIDS are vulnerable and at risk, given that HIV/AIDS in parents has been shown to increase paid child labour, increase poverty and create greater uncertainty about the future (Dayanandan, 2013; Kesby et al., 2006; Le Breton & Brusati, 2001; Richter, 2004; Russell & Seeley, 2010).

In the past, several studies had explored the association between paid child labour (paid market labour) and HIV/AIDS (Foster & Williamson, 2000; ILO, 2003; Le Breton & Brusati, 2001; Rau, 2002; Tumushabe, 2000) but social reproductive activities had been overlooked. Fortunately, there is an increase in research on children’s caring responsibilities in Africa resulting from wider research about the social impacts of HIV/AIDS especially in Sub-Saharan Africa. Eastern and Southern Africa have been disproportionately affected by HIV/AIDS justifying geographical focus on children’s care work in these regions (Evans, 2014). Despite this increased research, there is limited empirical research on factors that affect children’s social reproductive activities and the scale of children’s caring responsibilities in Africa (Evans, 2010; UNESCO, 2010). Evans (2014) highlights the need to quantify the extent, nature and outcomes of, children’s caring responsibilities in the global south.

Furthermore, children’s caring responsibilities are often neglected in official statistics yet the impact of HIV/AIDS on child labour begins within the houshold by impacting children’s involvement in social caring responsibilities including domestic labour within the hosuhold space (Bhalotra & Heady, 2003; Evans, 2010, 2014; FAO, IFAD, & ILO, 2011). Likewise, past research has mainly investigated the impact of HIV/AIDS on orphans even though the impact of adult illness on children starts when a parent is diagnosed as HIV-positive or when the parent is ill with HIV/AIDS, well before the parent dies (Alemtsehai & Tsegazeab, 2008; Dayanandan, 2013; Gilborn, Nyonyintono, Robert, & Jagwe-Wadda, 2001; Munyendo, Odera, Poipoi, & Mwanaongoro, 2013; Williams et al., 2003).

For the aforementioned reasons, this study seeks to fill the gap in empirical research by quantifying the nature of children’s social reproductive activities for children living in households that have a PLWHA (PLWHA households), compared to households without a known person living with HIV/AIDS (non-PLWHA households). The study investigates children’s work in terms of non-paid domestic work and non-paid family farm work and explores factors that impact on such work. For this study, children’s social reproductive activities in terms of non-paid family farm work and domestic work will be referred to as child work. Domestic work is a combination of household chores, child care, health care and personal care as elaborated below.

### Definition of child work

For this study, child work, also referred to as non-paid child work refers to children’s everyday work within the context of Africa. This includes household chores such as fetching water, firewood, cleaning and cooking; child care looking after children within the household; health care and personal care looking after sick members of the household including sick adults; family farm work for instance tending poultry, goats and cows, gardening and other family business; and in-kind-payment where payment is normally in terms of material items mostly food.

### Summary of study objectives

- To compare children’s participation in social reproductive activities for households with a known person living with HIV/AIDS (PLWHA) and households without a known PLWHA.
- Examine how different levels of factors including child and household-level factors impact participation in child work (domestic work and family farm work).
- Explore differences between factors that impact participation in non-paid domestic work and non-paid family farm work including gender roles.

The study examines how child work is influenced at different levels including child and household-level factors as illustrated in Evans (2014) (see Supplementary Material Figure-Table 2 for further illustration on factors that impact on children’s caring roles). In addition, the study includes children above 14 years old, who are usually treated as adults in most studies (including Demographic and Health Surveys (DHS), Living Standards Measurement Survey (LSMS), United Nations General Assembly Special Session (UNGASS) progress reports, Uganda National Household Surveys (UNHS) and UNAIDS reports) and are therefore excluded in child analysis despite being more vulnerable due to involvement in child work (Foster & Williamson, 2000). In this study, children are defined as 18 years or younger.

## Data and setting

### Survey methodology

This study uses household survey data from the 2010/2011 Centre for Health Economics Uganda HIV Survey (CUHS). The CUHS is a cross sectional survey that was undertaken from October 2010 to January 2011 in Uganda as part of PhD research. The survey data was collected through structured face to face interviews in which a questionnaire with a standardised set of questions was used. The region considered for the CUHS was Central Uganda, since approximately 40% of ART clients reside in or obtain their care from facilities located in the Central region (MOH, 2008). A summary of the survey methodology is described below; more detail is given elsewhere (Abimanyi-Ochom, Lorgelly, Hollingsworth, & Inder, 2013; Abimanyi-Ochom, Lorgelly, Inder, & Hollingsworth, 2012).

### Selection of ART service providers

The sample of households in the study was purposively selected from two major ART service providers based on the level of coverage and nature of services provided: The AIDS Support Organization (TASO) and Ministry of Health (MOH) Health Centers (HCs). MOH HCs fall under the government health department and only provide ART to PLWHA. In contrast, TASO, in addition to ART, provides social support through TASO’s Social Support Program that comprises services to mitigate the impact of HIV/AIDS on clients through sustainable livelihoods, child and nutritional support (TASO Uganda, 2008). MOH was selected given that it is the major provider of ART (ACP MOH, 2010) while TASO is one of the few ART service provider with considerable coverage that provides other support in addition to ART (TASO Uganda, 2008, 2015).

**Fig. 1.**
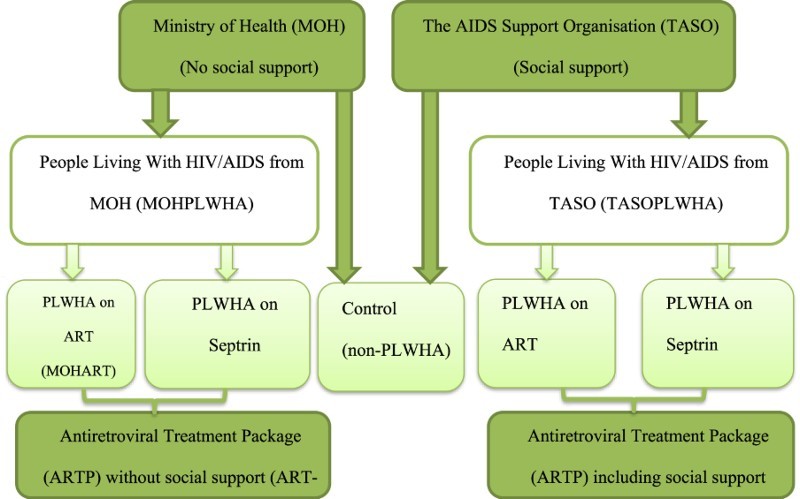
2010/2011 Centre for Health Economics Uganda HIV Survey sampling framework.

For both service providers, two levels of treatment were considered. Individuals on ART (ART group); and individuals on prophylactic septrin (waiting list group (awaiting ART)). We also selected a third comparator, households without a known PLWHA (non-PLWHA group) but residing in the same area as the TASO or MOH households (see Fig. 1). Non-PLWHA households were selected in the following manner to provide the best possible match in observable characteristics to PLWHA households: the second house (household) next to or opposite every third PLWHA household was approached to participate in the face to face structured interview, depending on availability and consent to participate in the survey. In the case of absence or refusal to participate, the next household was approached. The ART households and waiting list households are considered as the ‘combined PLWHA group’ since both of them have a PLWHA residing in them.

### Sampling strategy

Given that 79% of Ugandans live in rural areas (UBOS, 2013, 2016), 70% of the ART clinics sampled were in rural areas and the rest from urban areas. The total number of health clinics from which PLWHA were sampled for the survey was 34: 10 urban (29%) and 24 rural (71%).

The survey sample size was 596 households, comprising 79% (*N* = 450) PLWHA households (approximately half from TASO and half from MOH) and 21% (*N* = 146) non-PLWHA households from 11 districts in Central Uganda. Of the surveyed households, 126 (21%) were urban based and 470 (79%) were rural based.

### Study participants

Participants from TASO were selected from TASO’s enrolment list based on their receipt of social support in addition to ART in the last five years. TASO CDDPs Community ART Support Agents (CASAs) provided support to invite eligible TASO clients to participate in the study face to face structured interview. Similarly, MOH clients were selected from the ART register if they had been enrolled in the past five years. Selection from the MOH was random conditional on accessibility, being active and contact details for easy contact. For both service providers, PLWHA on prophylactic septrin were also selected from the enrolment list conditional on being accessible and not exceeding 5 years on septrin.

### Survey instrument and ethics

The survey instrument was a household and clinic questionnaire that looked at: chronic disease treatment packages (in this case ART packages for HIV/AIDS as a chronic disease); intra-household resource allocation and quality of life. The household questionnaire had been effectively used by International Food policy Research Institute in previous research in Eastern and Northern Uganda settings and had consistent results (hence proven reliable). The research assistants were trained through the questionnaire for two weeks. The survey instrument was pre-tested in communities with similar ART services to TASO and MOH for one day to ascertain reliability; the communities were culturally and linguistically relevant to our intended sample. Revision and correction of survey instrument was finalised during the training. The study was approved by the relevant participating organisations’ ethics committees- Monash University Human Research Ethics, The ministry of Health Uganda, The AIDS Support Organisation and Uganda National Council of Science and Technology (national ethics body in Uganda). Monash was the lead ethics approval organisation – ethics approval project Number: CF10/1036–2010000543. All participants provided a signed informed consent.

## Study theoretical underpinning

Research has revealed that children’s involvement in child work for children residing in households impacted by HIV/AIDS or disability is influenced by several factors at a number of levels. Firstly, children are impacted at the individual child level by their gender, age, birth order, personal attributes and co-residence with biological parent. Secondly, household-level factors such as the level of illness/disability of person needing care from the child, household socio-economic standing/power, disclosure of health status (especially for HIV/AIDS) and family structure changes impact children’s participation in child work. Thirdly community level factors especially access to formal and informal support through state welfare institutions, social support from the family and extended community support. Fourthly, social cultural values including community norms and expectations based on gender constructs, socio-cultural constructions of childhood and youth, and beliefs and awareness of disability/illness. Lastly, global issues including policies and legislations on the rights of children, migration issues, global incidence or prevalence of illness/disability, national socio-economic status and macro-related policy on the economy (Dayanandan, 2013; Evans, 2014; Evans & Becker, 2009; Evans & Skovdal, 2016; Robson, 2000; Robson et al., 2006; Skovdal et al., 2009, 2013). See Supplementary Figure-Table 2.

Given evidence of factors that impact on child work, our econometric model is as follow:

Child work = f (Individual child, Household, Community, Socio-cultural values and Global Policy environment).

Where Individual child are individual child-level factors; Household are household-level factors; Community are community level factors; Socio-cultural values include national policies and infrastructure factors in addition to social cultural norms; and Global Policy environment are global level factors. Note that for our study, socio-cultural and global factors were not controlled for in the analysis since these would not have any variation among children explored in this sample.

### Estimation strategy and variables

The data for this analysis is a sub-sample from the 2010/2011 CUHS. It comprises 1410 children aged 4–18 years old living at home during the school term from 452 households of which 349 households have a PLWHA, and the rest are non-PLWHA households. The analysis explores whether children from PLWHA households are more vulnerable to non-paid child work (in terms of domestic work, or family farm work separately and investigates factors that influence children’s non-paid labour allocation to both domestic and farm work.

There are two outcomes of interest (Dependent variables): children’s non-paid labour participation for two types of work: domestic and family farm work. The reference period for labour allocation is a typical week during the school term. Descriptive statistics illustrate how child work within the household space varies for children from PLWHA and non-PLWHA households; by gender, age differences and rural or urban residence. Child work outside the household space is also examined for comparison. This includes paid labour (3.4% of children *N* = 48) and non-paid labour (1.4% *N* = 20). However, multivariate regression analysis is not undertaken due to small numbers.

The key independent variable of interest is whether the child resides in a household with a PLWHA, was considered a child-level factor. Other control variables include individual (child) characteristics and household -level characteristics. Child-level factors include the age (age dummies; 0–4 years old, 6–12 years old, 13–18 years old) and gender of the child (girl), whether the child is enrolled in school (enrol), orphan status (orphan), whether the child’s mother resides in the household (mother co-resident) and whether the child had suffered from a disease for more than 6 months in the past 12 months prior to the survey (chronic disease).

Household-level characteristics include the age of the household head (Household head age); gender (male head); maximum educational attainment of household head (none, primary or secondary plus); religion (Muslim, Anglican, Catholic and other Christian); marital status of household head (married, widowed or separated); main occupation of household head (none, agricultural or non-agricultural); total number of children younger than five years old; total number of adult females in the household; household wealth index (high, average and low wealth); whether at least one person in the household has savings (got savings) or a loan^1^ (got loan); whether the household experienced at least one shock (shock) in the past 12 months (shocks include illness or death of a household member or relative, loss of a job by a household member or supportive relative, property loss to theft, farm loss due to harsh weather conditions, crops and livestock loss due to pests and diseases, and unfavourable market conditions including increased input prices and low output prices); whether the household owns land (own land); and type of residence (urban). Note that household religion can also fall under community level factors.

The household wealth index was constructed using a principal component analysis and comprises indicator variables including household assets (excluding ownership of land and financial assets to avoid collinearity) and utility services (McKenzie, 2005; Rutstein & Johnson, 2004; Vyas & Kumaranayake, 2006). The index was divided into thirds to represent top, average and low wealth.

Other variables used in the analysis include the child’s participation in either domestic work or farm work and severity of HIV/AIDS expressed as the CD4 cell count of the PLWHA or WHO-HIV stage (for the PLWHA only analysis) as a measure of level of illness. Severity of HIV/AIDS was not statistically significant (results not shown).

Bivariate model revealed that associations of gender of household head and WHO-HIV stage with non-paid child labour were not significant, hence these variables were excluded from the model.

### Empirical strategy and econometric issues

Associations between labour participation for child work (domestic and own farm work), PLWHA and the different child and household-level characteristics for children aged 4–18 years are investigated using bivariate and multivariate regression models using Stata 12.

#### Empirical model

Participation in non-paid child work is explored using a logistic regression model. The logistic model is a binary choice model that indicates whether or not a child participated in non-paid labour allocation for domestic work or farm work during a typical week of the school term.

The general model specification (using farm work as an example) is given below;(1)
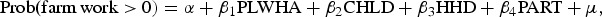
where, PLWHA is a child from a household with a person living with HIV/AIDS, CHLD are child-level variables, HHD are household-level variables, PART is the (alternate) labour participation variable (in this instance domestic work for Equations (1)), *α* is the constant term, 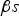
are the odds ratios and 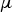 are the error terms.

Four models are used for family farm work and domestic work. Model 1 is a binary regression with PLWHA as the only regressor. Model 2 adds in child specific characteristics (PLWHA, CHLD), model 3 adds the household characteristics (PLWHA, CHLD and HHD) and model 4 adds the alternate child-work participation variable as a test for a substitution effect (PLWHA, CHLD, HHD and PART). All models are clustered at the household level and robust standard errors are obtained.

#### Econometric issues

The main explanatory variable (PLWHA) of interest is an indicator for whether or not a child is from a household with a person living with HIV/AIDS. There is a possibility of endogeneity bias in this coefficient, due to unobservable variables that are likely to affect both the likelihood of a person becoming HIV-positive and the likelihood that a child will engage in child work. Our analysis and methodology has taken several steps to minimise that bias. First, the survey sampling methodology ensured that the PLWHA households were ‘matched’ as much as possible to a neighbouring non-PLWHA household to minimise the differences in characteristics between the two groups (PLWHA and non-PLWHA households). Secondly, the models used in the analysis include a number of control variables – characteristics of the child and their household. The endogeneity bias that could remain must be due to omitted variables that would potentially affect both the likelihood of being HIV-positive and involvement in child work, after controlling for all these other observed characteristics. Given the set of controls, it is difficult to conceive of what unobserved factors these might be, or if they do exist, we argue their residual impact would be very small.

Consider one possible example of such an unobservable: suppose a child comes from a household where the adults have an attitude to life where they care little for the long-term consequences of their actions. Those with this kind of world view are more likely to engage in risk taking behaviour, and therefore may be more likely to be HIV-positive. This same attitude to life might also mean they care little about their child’s education, so they expect them to engage in more child work. Under this scenario, this unobserved attitude variable would be correlated with both the child-work variable and the PLWHA variable, leading to endogeneity bias. Recall that the child-work equation includes adult and child education measures, and indicators of household wealth, these variables would capture most of the effect of this unobserved attitude effect (e.g. those who are ‘short-sighted’ will invest little in their own or their child’s education, showing up in the education variable). The problem of unobservables may still remain, but it would be comparatively very small, capturing only the effect of attitude that does not show up in the observables like education and wealth.

To summarise, we would argue that any remaining bias due to unobservables is likely to be small or non-existent. At the same time, the possibility of bias does remain, and so, as is usual in analyses such as these, caution is needed about drawing causal rather than associative conclusions from the results.

## Results

### Descriptive statistics

Descriptive statistics summarising the survey sample according to their PLWHA status are presented in Table 2. As is common in many African cultures, results indicate that most children worked within their family even during the school term. The overall sample indicated that 51% and 81% of children participated in family farm and domestic work, respectively. The table shows that 53% of children from a household with a PLWHA participated in family farm work, significantly more than the 44% for non-PLWHA households. As expected, households with a PLWHA had a significantly higher incidence of orphans, children with a chronic disease and household heads that are widowed compared to non-PLWHA households (Table 2).

**Table 2.** Descriptive statistics of children 4–18 years and household-level characteristics.

Outcome variable	Overall sample (*N* = 1410)	PLWHA (*N* = 1098)	Non-PLWHA (*N* = 312)
Farm work participation	51.3%	**53.2%**	**44.5%**
Domestic work participation	80.6%	81.7%^+^	76.9%^+^
**Child individual characteristics**			
Age in years	10.9 (4.10)	10.9 (4.04)	10.9 (4.33)
Girl	52.1%	51.8%	53.2%
Enrolled in school	86.4%	86.5%	85.2%
Orphan	37.5%	**40.6%**	**26.8%**
Maternal orphan	10.2%	**11.3%**	**6.4%**
Mother resident in household	69.2%	68.9%	70.3%
Chronic disease	16.5%	**17.7%**	**12.3%**
**Household characteristics**	***N* = 452**	***N* = 349**	***N* = 103**
Household head age	44.5 (11.03)	44.3 (10.54)	45.0 (12.58)
Head married	43.4%	**37.8%**	**62.1%**
Head separated/divorce	16.1%	17.8%^+^	10.7%^+^
Head widowed	40.5%	**44.4%**	**27.2%**
Head agricultural occupation	59.7%	59.6%	60.2%
Head non-agricultural	35.4%	35.0%	36.9%
Head no occupation	2.0%	2.0%	1.9%
Head years of education	5.7% (3.62)	**5.4% (3.44)**	**6.5% (4.08)**
Head no education	58.8%	**62.5%**	**46.6%**
Head primary education	37.6%	35.5%	44.7%
Head secondary plus	4.4%	**2.9%**	**9.7%**
Number of adult females	1.4 (0.80)	1.5 (0.83)	1.3 (0.67)
Number of adult males	0.8 (0.82)	0.8 (0.83)^+^	1.0 (0.79)^+^
Number of adults	2.3 (1.18)	2.3 (1.19)	2.3 (1.13)
Number children <5 years old	0.8 (0.98)	0.8 (0.98)	0.9 (0.96)
Low wealth	27.4%	27.2%	28.2%
Average wealth	34.1%	34.4%	33.0%
High wealth	38.5%	38.4%	38.8%
Has savings	25.8%	25.3%	27.4%
Has loans	26.4%	**28.6%**	**18.6%**
Has experienced recent shock	85.6%	87.4%^+^	79.2%^+^
Owns land	65.5%	63.3%^+^	72.8%^+^
Urban residence	22.8%	**24.9%**	**15.5%**
Head Muslim	15.0%	14.9%	15.5%
Head Catholic	54.6%	54.4%	55.3%
Head Anglican	20.8%	20.1%	23.3%
Head other Christian	9.5%	10.6%	5.8%

Notes: Bold indicates **significant** difference between PLWHA and non-PLWHA at 95%; ^+^ *p* < .10; values are mean (SD = Standard deviation) or %. For tests of the mean, the *t*-test assumes equal means; for proportions, a chi squared test is performed.

Table 3 gives a summary of children’s social responsibilities for activities within the household space. Child-work activities include domestic work, family farm work, caring for the sick and child care. Children residing in non-PLWHA households spent more hours on domestic work (13 vs. 11 hours), family farm work (8 vs. 5 hours) and caring for the sick (4 vs. 3 hours) than children from PLWHA households. As expected from a patriarchal community, girls spent more time on domestic work (9 vs. 8 hours), looking after the sick (4 vs. 3 hours) and child care (4 vs. 3 hours) than boys. There was no evidence of boys engaging more in family work than girls. Older children had greater social responsibilities than younger children as evidenced in more hours of domestic work (10 vs. 8 hours), family farm work (7 vs. 5 hours) and caring for the sick (4 vs. 3 hours). Urban dwelling children spent more time caring for their younger siblings than rural-based siblings (5 vs. 3 hours). The values are small but difference in means of comparison groups are statistically different from zero at 5% level of significance.

**Table 3.** Descriptive statistics of child-work activities within the household space.

Work type-*N*, mean, 95% CI	PLWHA	NON-PLWHA	Girls	Boys	Older child	Younger child	Rural	Urban
Domestic work (*N* = 1137)	810	326	610	527	457	680	881	256
	**10.80** [10.25, 11.36]	**12.50** [11.15, 13.84]	**12.56** [11.70, 13.42]	**9.80** [9.16, 10.44]	**13.50** [12.48, 14.54]	**9.78** [9.19, 10.37]	**10.93** [10.34, 11.52]	**12.45** [11.12, 13.85]
Family farm work (*N* = 723)	527	195	356	367	330	393	628	95
	**4.87** [4.35, 5.38]	**7.48** [6.17, 8.80]	5.54 [4.70, 6.38]	5.60 [4.97, 6.23]	**6.83** [5.92, 7.74]	**4.51** [3.95, 5.07]	5.41 [4.86, 5.97]	6.58 [4.99, 8.17]
Looking after the sick (*N* = 350)	254	96	200	150	192	158	246	104
	**3.18** [2.71, 3.65]	**4.34** [3.30, 5.38]	**4.23** [4.70, 6.38]	**2.52** [2.17, 2.88]	**3.97** [3.40, 4.54]	**2.92** [2.23, 3.62]	3.31 [2.78, 3.84]	3.94 [3.11, 4.76]
Child care (*N* = 454)	321	132	284	170	223	231	331	123
	3.67 [3.30, 4.04]	4.25 [3.44, 5.06]	**4.40** [3.89, 4.90]	**2.89** [2.51, 3.27]	3.94 [3.43, 4.46]	3.72 [3.24, 4.21]	**3.42** [3.04, 3.81]	**4.93** [4.18, 5.69]

Notes: Bold indicates **significant** difference between PLWHA and non-PLWHA and older and younger child at 95%; older child refers to child aged 13–18 years old; values are *N*, mean hours and 95% CI in square brackets.

Considering children’s paid work outside the household, there is no difference in participation rates for children from PLWHA households and non-PLWHA households (3.19% and 3.85%, respectively). The average time spent in paid work for children from non-PLWHA households was significantly higher than that spent by children from PLWHA households (23 vs. 8 hours (see Table 4)). This is contrary to existing evidence which postulates that children from a household with an adult with HIV/AIDS are more likely to engage in the paid labour market than children from non-PLWHA households (Daniel, 2011; ILO, 2006).

**Table 4.** Descriptive statistics of child work outside the household space-paid work and non-paid (in-kind-payment) work.

Work type-*N*, mean, 95% CI	PLWHA	NON-PLWHA	Girls	Boys	Older child	Younger child	Rural	Urban
Paid work (*N* = 48)	19	29	10	38	41	7	33	15
	**7.89** [1.44, 14.35]	**22.69** [16.07, 29.31]	22.8 [8.20, 37.40]	15.26 [9.86, 20.67]	17.24 [11.66, 22.83]	14.43 [−0.80, 29.66]	14.91 [9.02, 20.79]	21.07 [10.59, 31.54]
In-kind-payment^a^ (*N* = 20)	8	12	7	13	14	6	15	5
	26.84 [1.13, 52.54]	23.17 [6.62, 39.71]	24.39 [−4.44, 53.21]	24.77 [8.87, 40.67]	**34.00** [17.96, 50.04]	**2.78** [0.89, 4.67]	19.47 [5.87, 33.06]	40.14 [0.36, 79.92]

Notes: Bold indicates **significant** difference between PLWHA and non-PLWHA and older and younger child at 95%; older child refers to child aged 13–18 years old; ^a^refers to non-paid work outside the household; values are *N*, mean hours and 95% CI in square brackets.

### Regression model results

#### Bivariate analysis

Table 5 displays bivariate relationships between the: (i) outcome variables; (ii) outcome variables and PLWHA, and (iii) outcome variables and child characteristics. Significant variables in the bivariate model are included in the multivariate analysis.

**Table 5.** Bivariate associations between the outcome variables, PLWHA and child-level characteristics.

	Farm work participation	PLWHA	Age	Gender (girl child)	Enrolled	Orphan	Chronic disease
**Logistic regression**							
Total-Labour participation OR [95% CI]	**–**	0.52 [0.18, 1.57]	1.05 [0.93, 1.18]	1.14 [0.88, 1.50]	0.64 [0.16–2.56]	1.12 (0.51, 2.43)	1.47 [0.53, 4.04]
Farm work participation OR [95% CI]	–	1.41^+^ [0.99, 2.01]	**1.17 [1.13, 1.21]**	**0.79 [0.64, 0.97]**	**2.10 [1.50–2.95]**	**1.84 (1.39, 2.42)**	1.21 [0.90, 1.63]
Domestic work participation OR [95% CI]	**10.66 [6.68–17.01]**	1.26 [0.86, 1.82]	**1.20 [1.14, 1.27]**	**1.38 [1.05, 1.81]**	**4.55 [3.20, 6.47]**	**1.91 (1.36, 2.68)**	1.43^+^ [0.94, 2.20]

Notes: Bold indicates **significant** at 95%; ^+^ *p* < .10; Odds Ratio and coefficients for logistic and OLS model, respectively; 95% CI in square brackets; total labour is aggregated labour that combines both domestic and farm work.

Results of the bivariate associations of non-paid child work and the PLWHA variable are presented in Model 1 of Tables 6 (Farm work) and 7 (domestic work). Children from PLWHA households were significantly more likely to participate in non-paid farm work than children from non-PLWHA households (UOR: 1.4, 95% CI: 1.0, 2.0) at 10% level of significance. Marginal analysis revealed that children from PLWHA households are 8.6 percentage points more likely to participate in farm work than children from non-PLWHA households (Supplementary Material-Table S1). Note that the association between outcome variables is significant, hence inclusion of the alternate child-work participation variable in multivariate model 4.

**Table 6.** Multivariate logistic model for farm work participation.

	(1) Bivariate	(2) CHLD	(3) HHD	(4) Other
	Logistic	Logistic	Logistic	Logistic
PLWHA	1.41^+^ [0.99–2.01]	1.35 [0.92, 1.98]	**1.71** [1.12, 2.60]	**1.84** [1.17, 2.89]
Child aged 6–12 years	** **	**10.98** [5.70, 21.15]	**11.64** [5.85, 23.16]	**6.06** [2.82, 13.01]
Child aged 13–18 years	** **	**15.17** [7.93, 29.03]	**16.05** [7.86, 32.77]	**8.85** [4.01, 19.56]
Girl child	** **	**0.71** [0.56, 0.89]	**0.83** [0.63, 1.09]	**0.74** [0.54, 1.00]
Enrolled in school	** **	1.43^+^ [0.96, 2.12]	1.47 [0.91, 2.37]	1.26 [0.77, 2.07]
Orphan	** **	**1.41** [1.04, 1.92]	**1.54** [1.01, 2.33]	**1.54** [1.01, 2.36]
Chronic disease		1.17 [0.86, 1.59]	1.39^+^ [0.98, 1.97]	1.26 [0.87, 1.83]
Mother resident			0.87 [0.60, 1.26]	0.84 [0.57, 1.25]
Head age			1.01 [0.99, 1.03]	1.01 [0.99, 1.03]
Head separated^a^			1.03 [0.60, 1.78]	0.93 [0.51, 1.70]
Head widowed			0.93 [0.56, 1.53]	0.87 [0.52, 1.46]
Head agricultural^b^			**3.71** [1.35, 10.18]	**4.77** [1.69, 13.46]
Head non-agricultural			2.70^+^ [0.94, 7.74]	**3.82** [1.29, 11.28]
Head primary			0.72^+^ [0.49, 1.04]	0.72 [0.48, 1.08]
Head secondary plus			1.93 [0.85, 4.36]	1.47 [0.62, 3.50]
Number adult females			1.13 [0.81, 1.57]	1.21 [0.87, 1.69]
Number children <5 years			0.94 [0.77, 1.15]	0.92 [0.75, 1.13]
High wealth^c^			**2.91** [1.70, 4.97]	**3.05** [1.71, 5.45]
Average wealth			**2.87** [1.72, 4.78]	**2.95** [1.73, 5.03]
Savings			1.28 [0.87, 1.89]	**1.66** [1.09, 2.53]
Experienced shock			1.16 [0.68, 1.99]	1.22 [0.70, 2.12]
Own land			**1.71** [1.15, 2.55]	**1.89** [1.23, 2.89]
Urban residence			0.60^+^ [0.35, 1.03]	**0.48** [0.28, 0.85]
Head Catholic^d^			0.93 [0.55,1.57]	0.89 [0.52,1.53]
Head Anglican			1.09 [0.57, 2.11]	0.98 [0.49, 1.95]
Head other Christians			0.63 [0.29, 1.35]	0.63 [0.29, 1.37]
Domestic work participation				**10.92** [6.55, 18.21]
Constant	0.80 [0.99, 2.01]	0.06 [0.03, 0.12]	0.002 [<0.01, 0.01]	<0.001 [<0.01, 0.001]
*N*	1410	1392	1253	1253

Notes: Bold indicates **significant** at 95%; ^+^ *p* < .10; Odds Ratios shown; 95% confidence intervals in square brackets; models clustered at household level.

^a^Base is married.

^b^Base is no occupation.

^c^Base is low wealth.

^d^Base is Muslim; *N* = Number of observations.

**Table 7.** Multivariate logistic model for domestic work participation.

	(1) Bivariate	(2) CHLD	(3) HHD	(4) Other
	Logistic	Logistic	Logistic	Logistic
PLWHA	1.26 [0.86, 1.82]	1.08 [0.71, 1.67]	1.01 [0.64, 1.59]	0.83 [0.51, 1.34]
Child aged 6–12 years	** **	**10.48** [6.75, 16.27]	**13.38** [8.12, 22.02]	**8.72** [5.01, 15.15]
Child aged 13–18 years	** **	**9.96** [6.23, 15.92]	**13.43** [7.72, 23.37]	**7.56** [4.01, 14.30]
Girl child		1.20 [0.86, 1.66]	1.30 [0.91, 1.87]	1.48^+^ [0.99, 2.21]
Enrolled in school	** **	**2.37** [1.59, 3.53]	**2.48** [1.59, 3.86]	**2.19** [1.37, 3.52]
Orphan		1.28 [0.88, 1.85]	1.27 [0.78, 2.07]	1.07 [0.62, 1.83]
Chronic disease		1.36 [0.89, 2.07]	1.39 [0.87, 2.22]	1.21 [0.72, 2.04]
Mother resident			0.97 [0.64, 1.48]	0.95 [0.60, 1.49]
Head age			1.02 [0.99, 1.04]	1.01 [0.99, 1.04]
Head separated^a^			1.33 [0.71, 2.49]	1.26 [0.60, 2.67]
Head widowed			1.31 [0.77, 2.23]	1.34 [0.74, 2.40]
Head agricultural^b^			0.30^+^ [0.08, 1.16]	**0.20** [0.05, 0.86]
Head non-agricultural	** **		0.27^+^ [0.07, 1.05]	**0.20** [0.05, 0.85]
Head primary			0.70^+^ [0.47, 1.06]	0.75 [0.48, 1.16]
Head secondary plus			2.27 [0.75, 6.88]	1.73 [0.54, 5.57]
Number adult females			0.81 [0.60, 1.10]	**0.75** [0.58, 0.97]
Number children <5 years			1.15 [0.91, 1.45]	1.25^+^ [0.98, 1.59]
High wealth^c^			1.36 [0.79, 2.36]	0.96 [0.52, 1.79]
Average wealth			1.59 [0.91, 2.79]	1.26 [0.69, 2.31]
Savings			**0.54** [0.33, 0.86]	**0.44** [0.27, 0.71]
Experienced shock			0.80 [0.42, 1.55]	0.73 [0.39, 1.39]
Own land			0.90 [0.57, 1.42]	0.70 [0.43, 1.13]
Urban residence			**1.98** [1.06, 3.68]	**2.66** [1.34, 5.28]
Hevad Catholic^d^			1.06 [0.63, 1.78]	1.14 [0.67, 1.94]
Head Anglican			1.28 [0.68, 2.39]	1.39 [0.70, 2.79]
Head other Christians			0.57 [0.24, 1.45]	0.74 [0.29, 1.88]
Farm participation				**11.05** [6.43, 18.98]
Constant	**3.72** [2.71, 5.13]	**0.24** [0.15, 0.42]	1.43 [0.06, 3.00]	0.97 [0.11, 8.00]
Observations	1410	1392	1253	1253

Notes: Bold indicates **significant** at 95% CI; highlighted is significant at 90% CI; ^+^ *p* <. 10; Odds Ratios shown; 95% confidence intervals in square brackets; models clustered at household level.

^a^Base is married.

^b^Base is no occupation.

^c^Base is low wealth.

^d^Base is Muslim; *N* = Number of observations.

#### Multivariate analysis

##### Differences between PLWHA and non-PLWHA households

Table 6 shows the results for participation in family farm work. Model 1 presents bivariate results explained above. As the child-level control variables are added in model 2, there is no difference between the households. In models 3 and 4, children from PLWHA households were almost twice as likely to participate in farm work compared to children from non-PLWHA households (AOR 1.84, 95% CI 1.17, 2.89). This implied that the difference between children from these two households cannot be attributed to observable differences in characteristics of the child or the household. In fact, with the full set of controls (model 4), the marginal effect of the PLWHA variable increased, suggesting a 10 percentage point greater likelihood of participation in family farm work for PLWHA households (Supplementary Material-Table S1). With an overall participation rate in family farm work of 51.3%, a 10 percentage point difference between PLWHA and non-PLWHA households is quite substantial.

Turning to domestic work, Table 7 showed little impact of being part of a PLWHA household on participation in such work. With participation rates of more than 80% overall, and even higher rates for school-aged children, this lack of differences was not surprising: effectively almost all children of a suitable age are involved in such work, whatever the household’s HIV status.

##### How child work varies with child characteristics

Table 6; Supplementary Material Table S1 and Table 7; Supplementary Material Table S2 (Table S2) show that, perhaps not surprisingly, older children (6–12 years old and 13–18 years old) were much more likely to participate in family farm work and domestic work than 4–5 year-olds. There is little difference between the participation rates of 6–12 year-olds and 13–18 year-olds, especially with domestic work (AOR 11, margins 28% and AOR 10, margins 27%, respectively): once a child is old enough to participate in such domestic chores, it appears virtually all children are expected to play a role.

Girls were significantly less likely to participate in family farm work than boys (Table 6; Table S1 AOR 0.74, margins 5% less) and there was some evidence that they were more likely to participate in domestic work (Table 7; model 4 Table S2).

Children enrolled in school were significantly more likely to participate in both family farm work and domestic work (8% and 7.8–10%, respectively). This is an interesting finding: enrolment in school would provide some indicator of the child’s positive engagement with their family and society, compared to a school-aged child who is not participating in school at all. Such positive engagement might show itself in a willingness to participate in family work, consistent with the positive participation effects of schooling.

Results in Table 6, Table S1 suggest that a child who is an orphan was significantly more likely to participate in family farm work compared to non-orphans (AOR 1.5, margins 7.2%, a similar result to previous research that has highlighted the vulnerability of orphaned children (Harms, Jack, Ssebunnya, & Kizza, 2010; Richter, 2004).

##### How child work varies with household characteristics

Model 3 in Tables 6 and 7; Tables S1 and S2 includes household variables. The inclusion of these household characteristics had little impact on the coefficients and statistical significance of the child-level characteristics, but offered some fresh insights into who were most vulnerable to child work.

One of the strongest variables impacting child participation in work is the household head’s primary occupation type. The base category here is no occupation, and compared to this category, if the household head worked in the agricultural or non-agricultural sector, the child was much more likely to be involved in family farm work (Tables S1,S2: around 26 percentage points more likely for those in the agricultural sector, and 22 percentage points more likely for non-agricultural employment). Notably, if the household head was employed, the child was also much less likely to be involved in domestic work (around 13–16 percentage points less likely). This did not fully compensate for the extra work in farm labour, but it did reduce the extra burden on the child.

There are some weak effects of household head’s education on child work, but no consistent or clear effects. This is interesting in itself, as it suggests parental education does not have a big impact on child work. This is in contrast to previous work, for example Ray (2000), where education level was negatively associated with all forms of child labour. Contrary to past studies, there was no evidence of increased child work due to the number of children younger than five years old in the household (Le Breton & Brusati, 2001; Moyi, 2011).

Table 6; Table S1 shows a very strong wealth effect: children from wealthier households were 18 percentage points more likely to participate in family farm work compared to children from the poorest category households (Table S1). As expected, owning land significantly encouraged family farm work participation for children by 11 percentage points (Table S1). Children residing in the urban area were less likely to participate in family farm work (12% less) (Table 6; Table S1) but 10% more likely to participate in domestic work (Table 7).

Having savings reduces children’s vulnerability through reduced domestic work participation (Table 7) but increased family farm work participation (Table 6). In most agrarian economies like Uganda, increased savings lead to increased investment in farming as a means of expanding farm land for both subsistence food production and cash crop production.

##### Participation in other work

Model 4 in Tables 6 and 7 and Table S1 and; S2 incorporates domestic participation in the family farm work model and family farm work participation in the domestic work model. There is very likely to be interaction between the different types of work, and ideally we would estimate a system of equations to capture this. However, the sample size was not adequate for this. Including the equivalent alternative outcome variable in each model provided some insight into the interactions, at the descriptive level at least.

Looking first at Table S1, even taking account of all the other factors, children who participated in domestic work were almost 39 percentage points more likely to also participate in family farm work. Conversely, children who participated in family farm work are 24 percentage points more likely to participate in domestic work (Table S2). These findings suggested that children’s family farm work participation and domestic work participation are complementary to each other.

### Robustness checks

#### Further analysis by gender

To investigate the possibility of gender differences in the effects on child work, the models were estimated separately for boys and girls (results available on request). Mostly, the results show very little variation with gender, except for a few key findings. First, boys residing in households with a PLWHA were twice as likely to participate in family farm work compared to boys from non-PLWHA households, a statistically significant difference.

Contrary to earlier research, school enrolment had no effect on participation in family farm work or domestic work for both boys and girls. This is possibly due to easy access to school through universal education. Free schooling reduced the opportunity cost of school enrolment when compared to fee-paying schooling, thereby reducing its impact on children’s time through non-paid labour including domestic and family farm work.

#### Controlling for HIV severity in PLWHA households

The models estimated to this point have not taken into account the severity of illness for adults who are HIV-positive. It is quite likely that the illness would affect intra-household behaviour very differently in the early stages of the disease compared to later when the illness has more severe effects on daily functioning. To explore this further, the models were re-estimated with the dummy variable for whether the household has a person living with HIV/AIDS replaced with the CD4 count of the person. The CD4 count is a reputable guide to severity. The results did not show any notable differences compared to the models reported in the paper.

## Discussion

The aim of this paper was to empirically explore children’s child work in terms of domestic work and family farm work for children residing in PLWHA households compared to those in non-PLWHA households; the study analysed how child-level and household-level factors were associated with children’s participation in child work.

Children residing in PLWHA households participated more in family farm work than children from non-PLWHA households; though mean farm work hours are marginally higher for children from non-PLWHA households. This is in contrast to earlier studies that revealed greater engagement of child work in PLWHA affected household (Lane et al., 2015; Robson et al., 2006). Our analysis by gender confirmed that boys from households affected by HIV/AIDS were twice as likely to engage in farm work.

Older children were more active in family farm work compared to the younger ones but no such trend for domestic work; possibly once a child is old enough to participate in such domestic chores, it appears virtually all children are expected to play a role. Other studies have noted age as a child-level factor that influences children’s level of caring responsibilities (Evans, 2014; Evans & Becker, 2009). Older children are indicated to be at a higher risk of abandoning school given that they are more physically mature and can take on more tasks (Foster & Williamson, 2000; Gillespie & Kadiyala, 2005; Moyi, 2011) and have been found to have greater levels of child-work relative to their younger siblings in virtually all categories of work (Fafchamps & Wahba, 2006).

This study confirmed expected gender roles within the household, consistent with past studies that pointed out girls to be more involved in domestic work (de Lange, 2009; Evans & Skovdal, 2016; Moyi, 2011; Robson, 2000; Tumushabe, 2000). Girls tend to shoulder most of the household chores and compensate for household labour loss, especially in the event of having a sick adult in the household (Foster & Williamson, 2000; Moyi, 2011; Yamano & Jayne, 2005). Analysis by gender confirmed earlier studies that emphasised greater involvement of boys in PLWHA households in farm work (FAO, IFAD, & ILO, 2011; Tumushabe, 2000; UNICEF, 2000). This is contrary to other research that found no such differences by gender roles within the household (Evans, 2014; Evans & Becker, 2009). Inconsistent with gendered constructions of care, our results indicated girls to have spent more hours in paid work compared to boys (7 hours more than boys per week). Evans (2012) noted that young men perceived their caring role predominantly in terms of providing financial support for themselves and their siblings, hence worked an average of 23 hours more per week than girls (Evans, 2012, 2014).

The finding that child work in terms of family farm labour increased even when the household head is not a farmer has been highlighted previously in the literature (Fafchamps & Wahba, 2006), but the compensating effect on domestic work has not, to our knowledge, been noted. This finding is independent of whether the household is HIV/AIDS affected or not because for most households, survival is dependent upon farming – Kaler, Alibhai, Kipp, Rubaale, and Konde-Lule (2010).

Education level as an indicator of socio-economic standing has been associated with reduced child labour in all forms (Ray, 2000) but our study revealed an inconsistent effect. Note that in our sample (Table 2), only 4.4% of household heads had secondary education, because the sample is mostly collected in poor, rural regions. So there may be insufficient variation in the sample to see the positive effects of parental education on child’s education (hence reducing child work). Similarly, wealth and savings as indicators of power would be expected to reduce child work but our results indicated differences within different types of child-work activities.

Greater wealth/savings protected children against domestic work but enhanced engagement in farm work. This has been explained in previous literature by the observation that wealthier families usually possess more agricultural land and are more likely to not only rely on hired labour but also depend on family labour (Barrows & Roth, 1990; Basu & Tzannatos, 2003; Bhalotra & Heady, 2003; de Lange, 2009; Jensen, 2000; Lastarria-Cornhiel & Melmed-Sanjak, 1999). This reaffirms the fact that the relationship between household wealth and child labour is paradoxical, especially in rural Africa: more wealth has been found to increase rather than reduce non-paid child-work hours (Bhalotra & Heady, 2003; de Lange, 2009). The wealth paradox has been explained in terms of land and labour market imperfections (Bhalotra & Heady, 2003) and the fact that child work has an inverted-U relationship with land wealth (Basu, Das, & Dutta, 2010). On the contrary, Becker’s study indicated low income as one of the factors that distinguished families with children involved in caregiving (Becker, 2007).

## Other factors, study limitations and strength

The dynamic nature of the HIV/AIDS epidemic has led to many changes in communities affected by HIV/AIDS (UNAIDS, 2013, 2016). As a result, other factors in addition to HIV/AIDS may have an important impact on children’s non-paid child work. These factors may include labour migration (of one of the household members) due to economic pressures or labour structure disintegration due to heightened HIV/AIDS deaths of young economically active individuals; improvement in adult health due to ART; lack of male support for unfavoured wives or widows and child labour support to the elderly (Schatz & Gilbert, 2012; Schatz, Madhavan, & Williams, 2011; UNAIDS, 2013). Our sample does not provide any evidence of more non-paid child labour participation for children residing with the elderly or widowed females. Factors beyond the child and household level also impact on children’s involvement in social caring responsibilities such as community level, socio-cultural and global factors (Evans & Becker, 2009).

The data used in this research has a number of limitations. In particular, the reliance on cross sectional data and estimates will inadequately capture important lifecycle effects like intergenerational persistence and harmful effects of non-paid child work within families (child labour trap) (Emerson & Souza, 2003). Future research using longitudinal data would be helpful in dealing with intergenerational concerns of child work. Longitudinal data will also capture the dynamic nature of children’s social responsibilities over time and space as highlighted in Evans (2014). Child-work data used in this study are self-reported by the adult in the household, hence a possibility of social desirability bias. The survey instrument was not statistically validated to ascertain accurate measurement of factors associated with households impacted by HIV/AIDS. Some of the control variables are very likely affected by the presence of a PLWHA in the household, therefore results are more applicable for associative conclusions.

A major strength of this study was the large sample size of children captured in engaging in child work (*N* = 1410) that made empirical analysis of children’s social responsibilities in terms of domestic work and farm work possible; given impact of HIV/AIDS. This is important given that a majority of children are engaged in these two work activities in Uganda and possibly most of East and South Sub-Saharan Africa, where the impact of the epidemic has been greatest. Additionally, data used in the analysis has a control group which minimises bias and makes comparison with the ordinary population possible.

## Conclusion, implications and recommendations for future research

This paper has quantitatively explored how child and household-level factors affect non-paid child work in both family farm work and domestic work for households with a PLWHA compared to non-PLWHA households.

The results suggest that children from households with a PLWHA participated more in child work related to farm work, more greatly for boys within affected households but no effect on girls. As a whole, girls were more engaged in domestic work and boys in farm work. This agrees with gendered social responsibilities within the household space in a patriarchal society like Uganda. The analysis revealed girls shared a greater proportion of providing financially for the household than boys by working more hours (on average) in paid work outside the household space, and thus children’s financial burden should not be associated with only boys. Age and being an orphan impacted participation in farm work but not domestic work, revealing differentials on how a child’s age and orphan status influence different work activities.

Furthermore, the study revealed that a household head’s occupation whether agricultural or non-agricultural increased children’s farm work participation but partially compensated children’s domestic work participation. Wealth had differential effects on different child-work activities; greater wealth led to greater involvement in farm work but reduced involvement in domestic work. Possession of savings is normally seen as a safety net against paid child labour but savings in this case infused greater participation in child work in terms of farm work. A focus on improving the socio-economic status of a household through wealth and savings should not be used as a ‘blanket solution’ to guarantee doing away with the negative outcomes of child work; differing perspectives and solutions need to be suggested within the context of the nature of child-work activity.

The findings suggest potential areas for action in programmes and policies. Strategies that can reduce the impact of farm labour deficits in households impacted by HIV/AIDS may reduce vulnerability of children from such households especially boys. Programme interventions to reduce children’s child work need to consider the nature of child-work activity to ensure effectiveness of such interventions. Improving socio-economic status through welfare assistance could be a means to reduce caring roles for children but contextual differences need to be noted to apply such policies in regions like Sub-Saharan Africa where the impact might be contrary to expectation; by increasing child work through farm work. Policy interventions need to consider gendered social responsibilities but must cater for exceptions to the rule such as protecting girls’ vulnerability through greater involvement in paid work outside the household space.

In conclusion, this study empirically demonstrates that boys residing in a household with a PLWHA, older children and orphans have a greater burden in farm work participation. Secondly, the research suggests that policies aimed at reducing household financial burdens need to also consider the girl child as the ‘breadwinner’ through her participation in paid work outside the household. Lastly, our findings have implications for poverty reduction policies and strategies-poverty reduction may not guarantee reduction in child work for family work in agrarian communities.

## Recommendations for future research

Child work exists in different spatial and temporal context hence future quantitative research needs to explore this contextual complexity of child work. Within the context of HIV/AIDS, further research needs to explore the temporal effect of formal safety nets such as welfare cash, non-cash support services and ART access on child work in the short and long term. There’s evidence that children’s caring responsibilities vary with time and space, hence it’s essential to further explore the dynamic nature of children responsibilities using longitudinal type data collected over time, preferably in national surveys. Cross section data gives a snapshot of the caring responsibilities at the time of the survey, but children’s caring responsibilities will vary due to fluctuations in HIV-related opportunistic infections and limitation in formal and informal safety nets. Such longitudinal data should capture the nature of child-work activities by differentiating work activities undertaken by children within the household space and outside the household space. There is also need to include quantitative measures to capture the different levels of factors that impact children’s degree of participation in child work to control for all confounders attributable to child work, especially at the community, socio-cultural and global/policy levels; more important for inter-community and international analyses.

## Supplementary Material

Final_Supplementary_Tables.docx
