# Supplementary material for: Invisible work: Child work in households with a person living with HIV/AIDS in Central Uganda
Source: SAHARA J. 2017 Oct 11;14(1):93–109. doi: 10.1080/17290376.2017.1379429 (PMC5639617; doi:10.1080/17290376.2017.1379429)
Supplement: Final_Supplementary_Tables.docx [file RSAH_A_1379429_SM4619.docx]

Supplementary Figure 1: Continuum of children’s caregiving

Source: Evans (2010, p.1480 and 2014, p.1896).
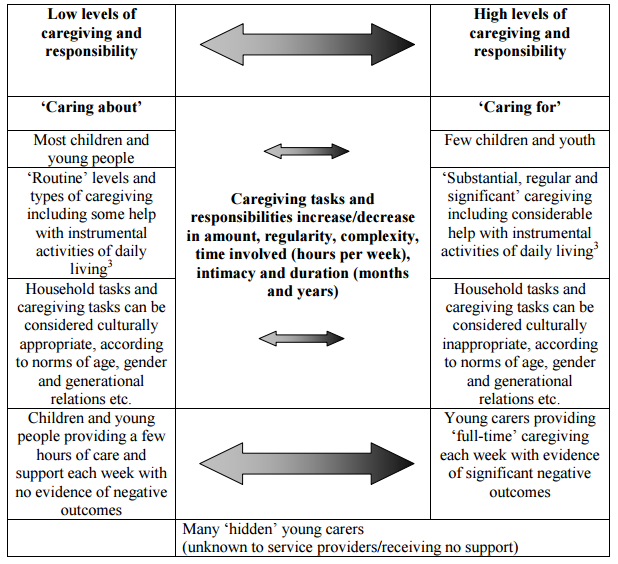


Supplementary Figure-Table 2: Risk and protective processes influencing whether children take on caring roles and the level of their involvement in care

| **Child** | **Household** | **Community** | **Socio-cultural** | **Global** |
| --- | --- | --- | --- | --- |
| Gender | Member cared for level of illness/disability | Access to extended family support | Gender norms and cultural constructions of care | Economic liberalisation and national socio-economic status |
| Age | Health status disclosure | Informal safety nets and social capital | Socio-cultural constructions of childhood and youth | Global prevalence and incidence HIV; inequality |
| Birth order | Socio-economic position/poverty status | Access to formal support | Stigma, beliefs and awareness of chronic illness/disability and ageing | Migration |
| Quality of relationship with cared for member | Changes in household/family structure |  |  | Implementation of child related rights conventions, commitment to address global inequalities |
| Personal attributes |  |  |  | Donor aid programs |
| Co-residence |  |  |  | National policies and legislations on children and families; social security and care systems; disability, caregivers and children; education… |

Source: Evan 2014, p.1909 and Evans and Becker 2009, p.229.

Table S1. Multivariate Logistic marginal effects for Farm work Participation

|  | (1)Bivariate | (2) CHLD | (3) HHD | (4) Other |
| --- | --- | --- | --- | --- |
|  | **Margins** | **Margins** | **Margins** | **Margins** |
| PLWHA | **0.086+** | 0. 066 | **0.099** | **0.100** |
|  | [-0.00,0.17] | [-0.18,0.15] | [0. 02,0.18] | [0.03,0.17] |
| child aged 6-12 years |  | **0.523** | **0.455** | **0.297** |
|  |  | [0. 38,0.66] | [0. 33,0.58] | [0.18,0.42] |
| child aged 13-18 years |  | **0.593** | **0.514** | **0.359** |
|  |  | [0.46,0.72] | [0.39,0.64] | [0.24,0.48] |
| Girl child |  | **-0. 075** | -0. 035 | **-0.050** |
|  |  | [-0.12,-0.03] | [-0.09,0.02] | [-0.10,-0.00] |
| Enrolled in School |  | **0. 078+** | 0.072 | 0.038 |
|  |  | [-0. 01,0.16] | [-0.02,0.16] | [-0.04,0.12] |
| Orphan |  | **0.076** | **0.079** | **0.072** |
|  |  | [0.01,0.14] | [0.00,0.16] | [0.00,0.14] |
| Chronic disease |  | 0.034 | **0.061+** | 0.038 |
|  |  | [-0.03,0.10] | [-0.00,0.13] | [-0.02,0.10] |
| Mother resident |  |  | -0.026 | -0.028 |
|  |  |  | [-0.09,0.04] | [-0.09,0.04] |
| Head Age |  |  | 0.003 | 0.002 |
|  |  |  | [-0.00,0.01] | [-0.00,0.01] |
| Head Separated**^∏^** |  |  | 0.006 | -0.012 |
|  |  |  | [-0.10,0.11] | [-0.11,0.09] |
| Head Widowed |  |  | -0.014 | -0.023 |
|  |  |  | [-0.11,0.08] | [-0.11,0.06] |
| Head agricultural**^†^** |  |  | **0.243** | **0.257** |
|  |  |  | [0.06,0.43] | [0.09,0.43] |
| Head non-agricultural |  |  | **0.184+** | **0.221** |
|  |  |  | [-0.01,0.38] | [0.04,0.40] |
| Head primary |  |  | **-0.062+** | -0.053 |
|  |  |  | [-0.13,0.01] | [-0.12,0.01] |
| Head secondary plus |  |  | 0.121 | 0.064 |
|  |  |  | [-0.03,0.27] | [-0.08,0.21] |
| Number adult females | |  | 0.022 | 0.031 |
|  |  |  | [-0.04,0.08] | [-0.02,0.09] |
| Number children<5yrs |  |  | -0.011 | -0.014 |
|  |  |  | [-0.05,0.03] | [-0.05,0.02] |
| High wealth**^$^** |  |  | **0. 198** | **0.184** |
|  |  |  | [0.10,0.29] | [0.09,0.28] |
| Average wealth |  |  | **0.195** | **0.178** |
|  |  |  | [0.11,0.29] | [0.10,0.26] |
| Savings |  |  | 0.046 | 0.083* |
|  |  |  | [-0.03,0.12] | [0.01,0.15] |
| Experienced shock |  |  | 0.028 | 0.033 |
|  |  |  | [-0.07,013] | [-0.06,0.12] |
| Own land |  |  | **0.100**** | **0.105** |
|  |  |  | [0.03,0.17] | [0.04,0.17] |
| Urban residence |  |  | **-0.095+** | **-0.120** |
|  |  |  | [-0.20,0.01] | [-0.21,-0.03] |
| Head Catholic**^#^** |  |  | -0.013 | -0.019 |
|  |  |  | [-0.11,0.08] | [-0.11,0.07] |
| Head Anglican |  |  | 0.016 | -0.003 |
|  |  |  | [-0.11,0.08] | [-0.12,0.11] |
| Head Other Christians |  |  | -0.087 | -0.077 |
|  |  |  | [-0.23,0.06] | [-0.21,0.05] |
| Domestic work participation | |  |  | **0.394**  [0.32,0.46] |
| Observations | 1410 | 1392 | 1253 | 1253 |

*Bold indicates* ***significant*** *at 95%; +p<.10; marginal effects shown; 95% confidence intervals in square brackets; Models clustered at household level; ^∏^base is married; ^†^base is No Occupation; ^$^base is low wealth; ^#^base is Muslim; N=Number of observations.*

Table S2. Multivariate marginal effects for logistic model for domestic work participation

|  | (1) Bivariate | (2) CHLD | (3) HHD | (4) Other |
| --- | --- | --- | --- | --- |
|  | **Margins** | **Margins** | **Margins** | **Margins** |
| PLWHA | 0. 045 | 0.021 | 0.014 | -0.006 |
|  | [-0. 01,0. 10] | [-0.03,0.07] | [-0.04,0.07] | [-0.06,0.04] |
| child aged 6-12 years |  | **0. 287** | **0. 301** | **0.222** |
|  |  | [0.24,0.33] | [0.25,0.35] | [0.17,0.27] |
| child aged 13-18 years |  | **0. 279** | **0.300** | **0.205** |
|  |  | [0.23,0.33] | [0.24,0.36] | [0.14,0.27] |
| Girl child |  | 0. 018 | 0.026 | **0.037+** |
|  |  | [-0.02,0.06] | [-0.02,0.07] | [-0.00,0.08] |
| Enrolled in School |  | **0.100** | **0.100** | **0.075** |
|  |  | [0.05,0.15] | [0.05,0.15] | [0.03,0.12] |
| Orphan |  | 0.034 | 0.034 | 0.013 |
|  |  | [-0.01,0.08] | [-0.02,0.09] | [-0.04,0.07] |
| Chronic disease |  | 0.036 | 0.038 | 0.019 |
|  |  | [-0.02,0.09] | [-0.02,0.09] | [-0.03,0.07] |
| Mother resident |  |  | -0.020 | -0.023 |
|  |  |  | [-0.07,0.03] | [-0.07,0.02] |
| Head Age |  |  | 0.002 | 0.001 |
|  |  |  | [-0.00,0.004] | [-0.00,0.004] |
| Head Separated**^∏^** |  |  | 0.024 | 0.016 |
|  |  |  | [-0.05,0.09] | [-0.06,0.09] |
| Head Widowed |  |  | 0.023 | 0.022 |
|  |  |  | [-0.04,0.09] | [-0.04,0.08] |
| Head agricultural**^†^** |  |  | **-0.113+** | **-0.141** |
|  |  |  | [-0.24,0.01] | [-0.26,-0.02] |
| Head non-agricultural |  |  | **-0.125+** | **-0.142** |
|  |  |  | [-0.25,0.00] | [-0.27,-0.02] |
| Head primary |  |  | -0.037 | -0.026 |
|  |  |  | [-0.08,0.01] | [-0.07,0.02] |
| Head secondary plus |  |  | 0.083 | 0.043 |
|  |  |  | [-0.06,0.22] | [-0.09,0.18] |
| Number adult females |  |  | -0.020 | **-0.026** |
|  |  |  | [-0.05,0.01] | [-0.05,-0.001] |
| Number children<5yrs |  |  | 0.011 | 0.017 |
|  |  |  | [-0.02,0.04] | [-0.01,0.04] |
| High wealth**^$^** |  |  | 0.031 | -0.012 |
|  |  |  | [-0.03,0.09] | [-0.08,0.05] |
| Average wealth |  |  | 0.049 | 0.017 |
|  |  |  | [-0.01,0.11] | [-0.05,0.08] |
| Savings |  |  | **-0.072** | **-0.085** |
|  |  |  | [-0.13,-0.02] | [-0.14,-0.03] |
| Experienced shock |  |  | -0.018 | -0.024 |
|  |  |  | [-0.09,0.05] | [-0.09,0.04] |
| Own land |  |  | -0.006 | -0.030 |
|  |  |  | [-0.06,0.05] | [-0.08,0.02] |
| Urban residence |  |  | **0.075** | **0.096** |
|  |  |  | [0.00,0.15] | [0.03,0.16] |
| Head Catholic**^#^** |  |  | 0.021 | 0.027 |
|  |  |  | [-0.04,0.08] | [-0.03,0.08] |
| Head Anglican |  |  | 0.048 | 0.052 |
|  |  |  | [-0.02,0.19] | [-0.02,0.12] |
| Head Other Christians |  |  | -0.063 | -0.033 |
|  |  |  | [-0.16,0.04] | [-0.12,0.06] |
| Farm participation |  |  |  | **0.246** |
|  |  |  |  | -0.006 |
| Observations | 1410 | 1392 | 1253 | 1253 |

*Bold indicates* ***significant*** *at 95%; +p<.10; marginal effects shown; 95% confidence intervals in square brackets; Models clustered at household level; ^∏^base is married; ^†^base is No Occupation; ^$^base is low wealth; ^#^base is Muslim; N=Number of observations.*

Table S2. Multivariate marginal effects for logistic model for combined domestic work participation

|  | (1) Bivariate | (2) CHLD | (3) HHD | (4) Other |
| --- | --- | --- | --- | --- |
|  | **Margins** | **Margins** | **Margins** | **Margins** |
| PLWHA | 0.034 | 0.010 | 0.001 | -0.191 |
|  | [-0.02,0.09] | [-0.04,0.06] | [-0.05,0.05] | [-0.67,0.29] |
| child aged 6-12 years |  | **0. 276** | **0. 291** | **0.216** |
|  |  | [0.23,0.32] | [0.24,0.34] | [0.17,0.27] |
| child aged 13-18 years |  | **0. 270** | **0.292** | **0.202** |
|  |  | [0.22,0.32] | [0.24,0.35] | [0.14,0.26] |
| Girl child |  | 0. 021 | 0.030 | **0.039+** |
|  |  | [-0.02,0.06] | [-0.01,0.07] | [-0.00,0.08] |
| Enrolled in School |  | **0.101** | **0.102** | **0.078** |
|  |  | [0.06,0.15] | [0.05,0.15] | [0.03,0.12] |
| Orphan |  | 0.029 | 0.027 | 0.006 |
|  |  | [-0.01,0.07] | [-0.03,0.08] | [-0.05,0.06] |
| Chronic disease |  | 0.036 | 0.037 | 0.019 |
|  |  | [-0.01,0.09] | [-0.02,0.09] | [-0.03,0.07] |
| Mother resident |  |  | -0.003 | -0.006 |
|  |  |  | [-0.05,0.04] | [-0.05,0.04] |
| Head Age |  |  | 0.002 | 0.001 |
|  |  |  | [-0.00,0.004] | [-0.00,0.004] |
| Head Separated**^∏^** |  |  | 0.032 | 0.023 |
|  |  |  | [-0.04,0.10] | [-0.05,0.10] |
| Head Widowed |  |  | 0.030 | 0.029 |
|  |  |  | [-0.03,0.09] | [-0.03,0.09] |
| Head agricultural**^†^** |  |  | **-0.134+** | **-0.158** |
|  |  |  | [-0.29,0.02] | [-0.30,-0.01] |
| Head non-agricultural |  |  | **-0.148+** | **-0.162** |
|  |  |  | [-0.30,0.01] | [-0.31,-0.02] |
| Head primary |  |  | -0.040 | -0.029 |
|  |  |  | [-0.09,0.01] | [-0.07,0.01] |
| Head secondary plus |  |  | 0.092 | 0.055 |
|  |  |  | [-0.03,0.22] | [-0.06,0.17] |
| Number adult females |  |  | -0.023 | **-0.029** |
|  |  |  | [-0.06,0.01] | [-0.05,-0.003] |
| Number children<5yrs |  |  | 0.016 | 0.022 |
|  |  |  | [-0.01,0.04] | [-0.01,0.05] |
| High wealth**^$^** |  |  | 0.035 | -0.004 |
|  |  |  | [-0.03,0.10] | [-0.07,0.06] |
| Average wealth |  |  | 0.052 | 0.023 |
|  |  |  | [-0.01,0.12] | [-0.03,0.08] |
| Savings |  |  | **-0.070** | **-0.083** |
|  |  |  | [-0.12,-0.02] | [-0.13,-0.03] |
| Experienced shock |  |  | -0.025 | -0.031 |
|  |  |  | [-0.10,0.05] | [-0.09,0.03] |
| Own land |  |  | -0.012 | -0.035 |
|  |  |  | [-0.06,0.04] | [-0.08,0.01] |
| Urban residence |  |  | **0.077** | **0.097** |
|  |  |  | [0.01,0.15] | [0.03,0.16] |
| Head Catholic**^#^** |  |  | 0.006 | 0.013 |
|  |  |  | [-0.05,0.06] | [-0.04,0.07] |
| Head Anglican |  |  | 0.027 | 0.033 |
|  |  |  | [-0.04,0.10] | [-0.04,0.10] |
| Head Other Christians |  |  | -0.060 | -0.030 |
|  |  |  | [-0.16,0.04] | [-0.12,0.06] |
| Farm participation |  |  |  | **0.239** |
|  |  |  |  | [0.19,0.29] |
| Observations | 1410 | 1392 | 1253 | 1253 |

*Bold indicates* ***significant*** *at 95%; +p<.10; marginal effects shown; 95% confidence intervals in square brackets; Models clustered at household level; ^∏^base is married; ^†^base is No Occupation; ^$^base is low wealth; ^#^base is Muslim; N=Number of observations.*
